# Supplementary material for: MetaRibo-Seq measures translation in microbiomes
Source: Nat Commun. 2020 Jun 29;11:3268. doi: 10.1038/s41467-020-17081-z (PMC7324362; doi:10.1038/s41467-020-17081-z)
Supplement: Supplementary file 10 — Supplementary Data 7 [file 41467_2020_17081_MOESM10_ESM.zip › File2/Confidence_High_Taxonomy/107652_out.krona.html]

Javascript must be enabled to view this page.

members
magnitude
magnitudeUnassigned
count
unassigned
taxon
rank

107652\_out

253

superkingdom
2
253

phylum
253
976

class
253
200643

order
253
171549

1
2005525
family

genus
1
375288

1
46503

SRS1041092\_contig\_number\_2779
species

family
815
4

genus
4
816


SRS017622\_contig\_number\_305
817
1
species

species

SRS015431\_contig\_number\_24393SRS045004\_contig\_number\_3322SRS103987\_contig\_number\_16379
3
821

171550
248
family


SRS013215\_contig\_number\_contig-100\_447.18081SRS013382\_contig\_number\_contig-100\_2083.2084SRS015065\_contig\_number\_26598SRS015369\_contig\_number\_contig-100\_9447.35835SRS016897\_contig\_number\_contig-100\_428.429SRS019601\_contig\_number\_contig-100\_1699.137245SRS022524\_contig\_number\_11216SRS045826\_contig\_number\_21876SRS047014\_contig\_number\_25906SRS065504\_contig\_number\_35885SRS074964\_contig\_number\_6876SRS075341\_contig\_number\_9299SRS1041091\_contig\_number\_contig-100\_1908.37340SRS1041143\_contig\_number\_5249SRS104636\_contig\_number\_25331SRS143417\_contig\_number\_20082SRS144183\_contig\_number\_contig-100\_4331.185371SRS146813\_contig\_number\_17531SRS147139\_contig\_number\_15509SRS893295\_contig\_number\_contig-100\_2998.2999
20
genus
248
239759


SRS054352\_contig\_number\_2554SRS063040\_contig\_number\_47489
2
1118061
species

328814
50

SRS011405\_contig\_number\_17412SRS013800\_contig\_number\_25246SRS014979\_contig\_number\_contig-100\_1395.186385SRS015782\_contig\_number\_36722SRS016381\_contig\_number\_2901SRS016989\_contig\_number\_14767SRS017701\_contig\_number\_18129SRS021484\_contig\_number\_27501SRS024132\_contig\_number\_48270SRS024435\_contig\_number\_31768SRS045244\_contig\_number\_2783SRS045713\_contig\_number\_20265SRS046502\_contig\_number\_11352SRS047044\_contig\_number\_contig-100\_844.102168SRS048164\_contig\_number\_30095SRS049773\_contig\_number\_32587SRS050941\_contig\_number\_20440SRS052697\_contig\_number\_38056SRS053335\_contig\_number\_17252SRS054905\_contig\_number\_5399SRS063985\_contig\_number\_23891SRS065176\_contig\_number\_13104SRS076976\_contig\_number\_9528SRS077194\_contig\_number\_21193SRS077392\_contig\_number\_33441SRS077589\_contig\_number\_3833SRS077641\_contig\_number\_23363SRS078176\_contig\_number\_14505SRS097920\_contig\_number\_14191SRS104084\_contig\_number\_9833SRS1041133\_contig\_number\_contig-100\_251.131403SRS1041140\_contig\_number\_contig-100\_313.123832SRS1041157\_contig\_number\_3058SRS105082\_contig\_number\_18388SRS1055043\_contig\_number\_25407SRS1055056\_contig\_number\_12046SRS1055067\_contig\_number\_9995SRS142923\_contig\_number\_3828SRS145497\_contig\_number\_18149SRS147766\_contig\_number\_6802SRS148159\_contig\_number\_49783SRS148319\_contig\_number\_15481SRS148784\_contig\_number\_21932SRS148970\_contig\_number\_4469SRS149181\_contig\_number\_18348SRS893252\_contig\_number\_6317SRS893279\_contig\_number\_11707SRS893358\_contig\_number\_10779SRS893378\_contig\_number\_11593SRS893383\_contig\_number\_contig-100\_2177.173841
species


SRS012273\_contig\_number\_12288SRS013476\_contig\_number\_contig-100\_1872.222703SRS013951\_contig\_number\_36164SRS014683\_contig\_number\_13821SRS014855\_contig\_number\_5471SRS015190\_contig\_number\_13693SRS015663\_contig\_number\_15556SRS015794\_contig\_number\_contig-100\_59.94142SRS015890\_contig\_number\_21631SRS016095\_contig\_number\_contig-100\_3208.124462SRS016267\_contig\_number\_11191SRS016495\_contig\_number\_contig-100\_2625.115835SRS016629\_contig\_number\_639SRS017247\_contig\_number\_9463SRS018313\_contig\_number\_11740SRS018656\_contig\_number\_11254SRS018817\_contig\_number\_5079SRS018984\_contig\_number\_29733SRS019161\_contig\_number\_contig-100\_493.242776SRS019267\_contig\_number\_6795SRS019286\_contig\_number\_contig-100\_479.49612SRS019787\_contig\_number\_12099SRS019968\_contig\_number\_16442SRS024075\_contig\_number\_contig-100\_254.109140SRS024492\_contig\_number\_16445SRS042284\_contig\_number\_6592SRS043768\_contig\_number\_32349SRS046369\_contig\_number\_13358SRS048870\_contig\_number\_contig-100\_2776.119016SRS049900\_contig\_number\_16969SRS050925\_contig\_number\_22860SRS053398\_contig\_number\_contig-100\_1032.154926SRS053573\_contig\_number\_2681SRS054590\_contig\_number\_contig-100\_529.25567SRS057480\_contig\_number\_11088SRS063813\_contig\_number\_452SRS064232\_contig\_number\_2861SRS064557\_contig\_number\_contig-100\_867.81272SRS075773\_contig\_number\_contig-100\_652.221394SRS076929\_contig\_number\_contig-100\_511.144510SRS078242\_contig\_number\_19360SRS098655\_contig\_number\_13104SRS100021\_contig\_number\_10791SRS1041032\_contig\_number\_14731SRS1041036\_contig\_number\_5745SRS1041137\_contig\_number\_32766SRS104327\_contig\_number\_4289SRS1054716\_contig\_number\_14689SRS142712\_contig\_number\_1536SRS142890\_contig\_number\_38775SRS144297\_contig\_number\_12191SRS144362\_contig\_number\_17411SRS144537\_contig\_number\_22260SRS146764\_contig\_number\_4140SRS146812\_contig\_number\_55753SRS147271\_contig\_number\_37076SRS147346\_contig\_number\_44601SRS147557\_contig\_number\_24458SRS893369\_contig\_number\_contig-100\_41.56075
1262694
59
species

species

SRS011134\_contig\_number\_51740SRS012849\_contig\_number\_7996SRS013965\_contig\_number\_35494SRS014313\_contig\_number\_4619SRS014459\_contig\_number\_contig-100\_418.223060SRS014613\_contig\_number\_20251SRS015217\_contig\_number\_30339SRS015694\_contig\_number\_22393SRS015854\_contig\_number\_21887SRS016056\_contig\_number\_1241SRS016335\_contig\_number\_40621SRS018351\_contig\_number\_17315SRS018541\_contig\_number\_538SRS018575\_contig\_number\_7375SRS019030\_contig\_number\_16942SRS019068\_contig\_number\_59573SRS019808\_contig\_number\_18552SRS022071\_contig\_number\_13611SRS024388\_contig\_number\_1009SRS024549\_contig\_number\_5535SRS043411\_contig\_number\_12048SRS045645\_contig\_number\_23328SRS050299\_contig\_number\_4785SRS050998\_contig\_number\_17027SRS051610\_contig\_number\_8630SRS051882\_contig\_number\_19666SRS056273\_contig\_number\_22164SRS056519\_contig\_number\_9460SRS058070\_contig\_number\_13420SRS058723\_contig\_number\_7886SRS062654\_contig\_number\_16138SRS062701\_contig\_number\_5036SRS063489\_contig\_number\_5183SRS063518\_contig\_number\_24510SRS064276\_contig\_number\_41851SRS064645\_contig\_number\_842SRS075878\_contig\_number\_1917SRS075984\_contig\_number\_21320SRS077231\_contig\_number\_24193SRS077335\_contig\_number\_2425SRS077454\_contig\_number\_16918SRS077552\_contig\_number\_22530SRS097958\_contig\_number\_15155SRS098717\_contig\_number\_15749SRS1041142\_contig\_number\_8476SRS1041145\_contig\_number\_24307SRS1041164\_contig\_number\_2990SRS104912\_contig\_number\_21015SRS1055022\_contig\_number\_21323SRS1055069\_contig\_number\_11822SRS1055083\_contig\_number\_13493SRS140513\_contig\_number\_12592SRS142542\_contig\_number\_19983SRS143523\_contig\_number\_21913SRS144603\_contig\_number\_14833SRS144753\_contig\_number\_contig-100\_890.77101SRS147653\_contig\_number\_2990SRS147919\_contig\_number\_19677SRS148253\_contig\_number\_2089SRS148511\_contig\_number\_12942SRS148817\_contig\_number\_8830SRS892882\_contig\_number\_12126SRS893172\_contig\_number\_13466SRS893259\_contig\_number\_4849SRS893327\_contig\_number\_9895
214856
65

species

SRS013638\_contig\_number\_16969SRS016018\_contig\_number\_1780SRS043841\_contig\_number\_contig-100\_163.79628SRS044535\_contig\_number\_21665SRS049959\_contig\_number\_45573SRS098571\_contig\_number\_45898SRS1041033\_contig\_number\_29641SRS1041118\_contig\_number\_15366
1896969
8


SRS013940\_contig\_number\_21926SRS018133\_contig\_number\_8235SRS022137\_contig\_number\_17523SRS055982\_contig\_number\_19254SRS065397\_contig\_number\_11232SRS076756\_contig\_number\_1518SRS077086\_contig\_number\_14230SRS098644\_contig\_number\_contig-100\_2542.277364SRS101433\_contig\_number\_13716SRS143148\_contig\_number\_14843SRS143181\_contig\_number\_contig-100\_2608.96689
28117
11
species


SRS043701\_contig\_number\_16285
1
1872444
species

1
2292911

SRS016840\_contig\_number\_contig-100\_628.34007
species

1896970
2

SRS148721\_contig\_number\_20490SRS149075\_contig\_number\_3906
species


SRS018623\_contig\_number\_12439SRS021948\_contig\_number\_22005SRS042628\_contig\_number\_36418
1946007
3
species

2292910
12

SRS011084\_contig\_number\_32630SRS015264\_contig\_number\_5300SRS023715\_contig\_number\_3000SRS074586\_contig\_number\_4910SRS075078\_contig\_number\_26607SRS077024\_contig\_number\_6189SRS1041129\_contig\_number\_13343SRS105153\_contig\_number\_20014SRS147425\_contig\_number\_10760SRS893230\_contig\_number\_2708SRS893341\_contig\_number\_15963SRS971275\_contig\_number\_9577
species

6
1263035

SRS023526\_contig\_number\_11866SRS054956\_contig\_number\_15683SRS075821\_contig\_number\_19028SRS098061\_contig\_number\_4023SRS1055099\_contig\_number\_6420SRS149784\_contig\_number\_24540
species

species
1499682
3

SRS053214\_contig\_number\_4253SRS1041136\_contig\_number\_7489SRS1041138\_contig\_number\_12915

species

SRS143876\_contig\_number\_14421
1288121
1

species

SRS049402\_contig\_number\_10435SRS140492\_contig\_number\_4442
2
1946009

2
328813

SRS015579\_contig\_number\_22024SRS143722\_contig\_number\_11655
species
